# The lncRNA NEAT1/miRNA-766-5p/E2F3 Regulatory Axis Promotes Prostate Cancer Progression

**DOI:** 10.1155/2022/1866972

**Published:** 2022-02-21

**Authors:** Wenhui Zhao, Xinshu Zhu, Qiu Jin, Bo Lin, Runyuan Ji

**Affiliations:** Department of Pathology, Huai'an Key Laboratory of Gastric Cancer, Jiangsu College of Nursing, Huai'an, Jiangsu 223001, China

## Abstract

**Background:**

Prostate cancer (PCa) is one of the most common malignancies in men. Increasing evidence has demonstrated that dysregulation of long noncoding RNAs (lncRNAs) is closely related to carcinogenesis and cancer progression. lncRNA NEAT1 has recently been identified as a carcinogenic regulator of multiple cancers; however, the role of NEAT1 on PCa is still poorly understood.

**Methods:**

Kaplan–Meier was conducted to determine the overall survival rate in PCa patients with aberrant NEAT1 levels. qRT-PCR analysis was performed to detect expressions of NEAT1 and miR-766-5p in tissues and cells. In addition, CCK-8, colony formation, flow cytometry analysis, wound healing, and transwell assay were conducted to determine cell proliferation, cell arrest, apoptosis, migration, and invasion. The western blot assay was utilized to determine E2F3 and cell growth-related proteins. The relationship between NEAT1 and miR-766-5p or miR-766-5p and E2F3 was verified by correlation analysis and dual-luciferase reporter assay.

**Results:**

Here, we find that NEAT1 is overexpressed in PCa tissues and cell lines. Besides, silencing of NEAT1 inhibits cell proliferation, invasion, and migration and promotes cell apoptosis and cell cycle arrest. Further mechanistic studies find that NEAT1 sponges miR-766-5p, and miRNA-766-5p is negatively correlated with the expression of NEAT1. In addition, the functional experiment shows that upregulation of miRNA-766-5p inhibits PCa proliferation, migration, and invasion. Furthermore, E2F transcription factor 3 (E2F3) is testified to be the downstream target gene of miRNA-766-5p. Finally, the rescue experiment revealed that miRNA-766-5p inhibition largely restores NEAT1 downregulation-mediated function on PCa progression, while E2F3 knockdown partly removes the effects of miRNA-766-5p inhibitor.

**Conclusions:**

In conclusion, NEAT1 facilitates PCa progression by targeting the miRNA-766-5p/E2F3 axis.

## 1. Introduction

As one of the most common malignant tumors in the male urinary system, prostate cancer (PCa) is a malignant tumor occurring in the prostatic epithelium [1]. According to the latest world epidemiological statistics in 2019, the morbidity of PCa has surpassed that of lung cancer, ranking the first (20%, 174,560 cases), and the mortality rate is the second (10%, 31,620 cases) [2]. Although the incidence of PCa in China is lower than that in Europe and the United States, the incidence of PCa has been on the rise in recent years [3]. Although much progress has been made in diagnosing and treating PCa, the long-term prognosis is still poor [4, 5]. So, new molecular mechanisms of PCa progression need to be explored urgently to design more effective treatment strategies.

lncRNA is a kind of RNA molecule with a length of more than 200 nucleotides that have no coding ability or encodes only a short polypeptide [6, 7]. lncRNAs were previously considered a transcription by-product of cellular processes. However, with the development of epigenetics, people gradually realize its important biological role, finding that lncRNAs are involved in various cellular processes, including apoptosis, differentiation, and proliferation [8–10]. Accumulating research studies reveal that lncRNAs play an important role in accelerating or inhibiting tumor progression. According to the previous studies, lncRNAs regulate miRNAs expression in competitive endogenous RNAs (ceRNAs) form [11]. For instance, lncRNA SNHG14 promotes the tumorigenesis of PCa via targeting miR-5590-3p to regulate YY1 expression [12]. For another example, knockdown of lncRNA LOXL1-AS1 inhibits the occurrence and development of PCa via the miR-541-3p/CCND1 axis [13].

The oncogenic role of lncRNA NEAT1 had been reported in multiple cancers. However, the understanding of the regulatory role of NEAT1 in PC was very limited, so it was urgent to study and clarify the mechanism of NEAT1. Our study mainly elucidated the possible usefulness of NEAT1 on mediating PCa progression, providing a novel promising goal for PCa treatment.

## 2. Methods

### 2.1. Clinical Samples

This study obtained a total of 50 paired PCa tumor tissues and adjacent nontumor tissues from Jiangsu College of Nursing. The experimental protocol was established according to the ethical guidelines of the Helsinki Declaration and was approved by the Ethics Committee of Jiangsu College of Nursing. All participants had signed informed consent forms.

### 2.2. Expression of NEAT1 in GEO Database

The expression of NEAT1 in prostate cancer tumor and adjacent nontumor tissues was obtained from Gene Expression Omnibus (GEO). The original data of GSE29079 were downloaded and analyzed by GEO 2R.

### 2.3. Cell Culture and Transfection

RWPE-1 (human normal prostatic epithelial cells) and PC3, P4E6, LNCaP, and DU145 (PCa cell lines) were obtained from the Chinese Type Culture Collection (CTCC, Shanghai, China). All cells were cultured in DMEM containing 10% fetal bovine serum (FBS) and incubated at 5% CO_2_ at 37°C.

sh-NEAT1 (shRNA-NEAT1-1, 5′-GTGAGAAGTTGCTTAGAAA-3′ and shRNA-NEAT1-2, 5′-TGGTAATGGTGGAGGAAGA-3′), sh-E2F3 (sh-E2F3-1, 5′-ACCGCCAAGACCACAATGGGAATATCTCGAGATA-3′ and sh-E2F3-2, 5′-AAAACCAAGACCACAATGGGAATATCTCGAGATA-3′), miR-766-5p mimic (5ʹ-CGACUCCGAUUCUUGUUAGAG-3ʹ), and miR-766-5p inhibitor (5ʹ-AAGACCAGCACCAAUUCCUCCU-3ʹ) were designed and purchased from GenePharma (Shanghai, China). These vectors were transfected into cells using Lipofectamine 2000 reagent (Invitrogen, Shanghai, China) according to the manufacturer's instructions.

### 2.4. CCK-8 Assay

Briefly, cells were placed in the 96-well plate with a density of 3 × 10^4^ cells/well and then added CCK-8 (10 *μ*L) reagent into each well for 2 h at 37°C. The optical density (OD) value was measured at 450 nm by using an ultraviolet spectrophotometer (Shimadzu, Kyoto, Japan).

### 2.5. qRT-PCR

RNA was extracted from cell and tissue samples using TRIzol reagents (Takara, Otsu, Shiga, Japan). The PrimeScript^TM^ II 1st Strand cDNA Synthesis Kit (Takara, Otsu, Shiga, Japan) was used to induce cDNA. The protocol for RT was as follows: 37°C for 15 min, 85°C for 5 s, and termination at 4°C. Quantitative real-time quantitative PCR (qRT-PCR) was performed using SYBR® green main mixture in 4800 real-time PCR instruments (Bio-Rad, CA, USA). PCR thermocycling conditions were as follows: predenaturation at 95°C for 30 s, followed by 40 cycles of denaturation at 95°C for 5 s, and annealing at 60°C for 30 s. The primers for qRT-PCR are shown in Table 1.

### 2.6. Western Blot Assay

Total proteins were extracted from cells using lysis buffer, and the concentrations of proteins were measured by the BCA method. 12% SDS-PAGE was utilized to separate proteins and then transferred onto PVDF membranes. Then, 5% skimmed milk was used to block membranes for 50 min at room temperature followed by incubation with primary antibodies at 4°C overnight. The primary antibodies are listed as follows: anti-cyclin D1 (1 : 1000; ab16663; Abcam, Shanghai, China), anti-p21 (1 : 1000; ab109520; Abcam, Shanghai, China), anti-Bax (1 : 1000; ab32503; Abcam, Shanghai, China), anti-Bcl-2 (1 : 1000; ab32124; Abcam, Shanghai, China), anti-cleaved-caspase-3 (1 : 1000; ab2302; Abcam, Shanghai, China), anti-cleaved-caspase-9 (1 : 1000; ab2324; Abcam, Shanghai, China), anti-Cox-2 (1 : 1000; ab179800; Abcam, Shanghai, China), anti-MMP-2 (1 : 1000; ab92536; Abcam, Shanghai, China), anti-MMP-9 (1 : 1000; ab76003; Abcam, Shanghai, China), anti-E2F3 (1 : 1000; ab152126; Abcam, Shanghai, China), and anti-GAPDH (1 : 1000; ab8245; Abcam, Shanghai, China). The membranes were then incubated with secondary antibody (1 : 1000; ab7090; Abcam, Shanghai, China) on the next day at 37°C for 2 h. An enhanced chemiluminescence kit and the ImageJ software were applied to protein quantification.

### 2.7. Colony Formation Test

Logarithmic growth cells were inoculated into a 6-well plate. When visible clones appeared in the Petri dish, the culture was terminated. After discarding the supernatant, 4% paraformaldehyde was fixed, and GIMSA staining solution was dyed for 10–30 min. Finally, cell clone numbers were counted under a microscope (Optical-SH, Shanghai, China).

### 2.8. Wound Healing Assay

Firstly, cells were seeded into the 6-well plate until they generated a confluent monolayer. Then, using a sterile pipette tip, a scratch was made in the confluent cells. After 48 h, the scratch-induced wound was observed under a microscope and analyzed by ImageJ.

### 2.9. Transwell Analysis

According to the previous study, the migration and invasion of PCa cells were detected by the transwell assay [14]. 100–150 *μ*l of cell suspension was added to the upper chamber of the transwell with or without precoated with Matrigel (BD Bioscience, Beijing, China) for the invasion and migration assay, respectively. Meanwhile, 600–800 *μ*l of medium containing 10% serum was added to the lower chamber, and the medium was incubated at room temperature for 48 h. Then, the medium was transferred to a well prefilled with approximately 800 *μ*l of methanol and fixed for 30 min at room temperature. The chamber was removed, the upper chamber fixative was aspirated, transferred to wells prefixed with approximately 800 *μ*l Giemsa staining solution, and stained for 15–30 min at room temperature. The cells were carefully wiped on the bottom membrane surface of the upper chamber with a wet cotton swab. Random fields of view were taken under the microscope for counting, and the results were tallied.

### 2.10. Flow Cytometry

For apoptosis, cells were collected and suspended with 100 *μ*L of binding buffer. Next, FITC annexin V and propidium iodide (PI) were used to stain cells in the dark. After 10 min, 400 *μ*L of binding buffer was added to each tube, and the apoptosis was assessed by flow cytometry (BD Bioscience, CA, USA). For the cell cycle, to put it simply, the cells were fixed with 70% ethanol, digested with RNase for 30 min, stained with propidium iodide, and incubated for 15 min. Finally, flow cytometry was performed to measure the cell cycle.

### 2.11. Luciferase Reporter Assay

A luciferase reporter assay was performed to detect the relationship between NEAT1 and miRNA-766-5p or between miRNA-766-5p and E2F3. Briefly, full-length of NEAT1 or E2F3 was synthesized by PCR amplification and inserted into the pGL3-Basic vector to construct wild-type NEAT1 (NEAT1-WT) or wild-type E2F3 (E2F3-WT). Meanwhile, the mutant type of NEAT1 (NEAT1-Mut) or E2F3 (E2F3-Mut) was designed by GenePharma (Shanghai, China). Then, these vectors were cotransfected with NC mimic or miR-766-5p mimic into LNCaP and PC3 cells using Lipofectamine 2000 reagent (Invitrogen, Shanghai, China) as per the manufacturer's instructions. Finally, the luciferase activity was measured by using the dual-luciferase assay kit (Zeye, Shanghai, China).

### 2.12. RNA-Binding Protein Immunoprecipitation (RIP) Assay

The RIP experiment was carried out with the Magna RIP kit (Magna, ON, CAN). Briefly, the lysed cells were incubated in the RIP buffer solution, and the magnetic beads were labeled with anti-Ago2 or IgG. The abundance of NEAT1 and miRNA-766-5p was verified by qRT-PCR.

### 2.13. Statistical Analysis

Data were presented as the mean ± standard deviation (SD). Comparisons were determined using the unpaired Student's *t*-test and one-way ANOVA followed by Bonferroni's post hoc test. *P* < 0.05 indicated statistically significant.

## 3. Results

### 3.1. NEAT1 Was Highly Expressed in PCa Tissue and Cell Lines

**Figure 1:**
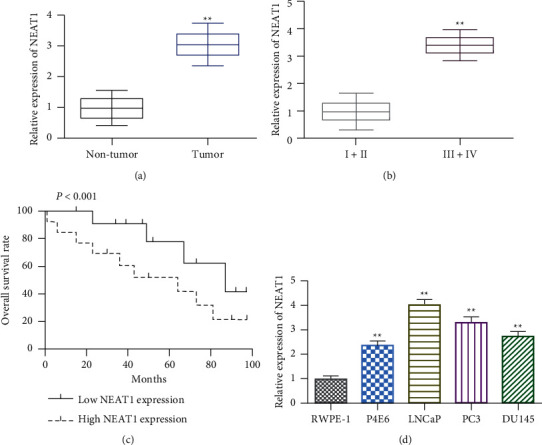
NEAT1 was highly expressed in PCa tissues and cell lines. (a) The levels of NEAT1 in PCa tumor tissues compared with nontumor adjacent tissues. ^*∗∗*^*P* < 0.01, tumor vs. nontumor. (b) The levels of NEAT1 in PCa patients with stage I + II and stage III + IV assessed by qRT-PCR. ^*∗∗*^*P* < 0.01, III + IV vs. I + II. (c) Survival analysis based on PCa patients with high-level or low-level NEAT1. (d) The level of NEAT1 in PCa cell lines. ^*∗∗*^*P* < 0.01, P4E6, LNCaP, PC3, and DU145 vs. RWPE-1.

We firstly measured the levels of NEAT1 by qRT-PCR analysis. As shown in Figure 1(a), NEAT1 was overexpressed in PCa tissues compared to adjacent normal tissues. More interestingly, we found that the level of NEAT1 in PCa patients with tumor stage III + IV was higher than that of stage I + II (Figure 1(b)). This result is consistent with the gene expression profiles obtained from the Gene Expression Omnibus (GEO) database (GSE29079) (Figure S1). Meanwhile, the Kaplan–Meier curve showed that the survival rate of the low-level NEAT1 group was superior to that of the high-level group (Figure 1(c)). In addition, compared with RWPE-1, NEAT1 was highly expressed in PCa cell lines, especially in LNCaP and PC3, which were chosen for the following experiments. Altogether, these results indicated the carcinogenic role of NEAT1 in PCa.

### 3.2. Knockdown of NEAT1 Inhibited Cell Growth in Prostate Cancer

**Figure 2:**
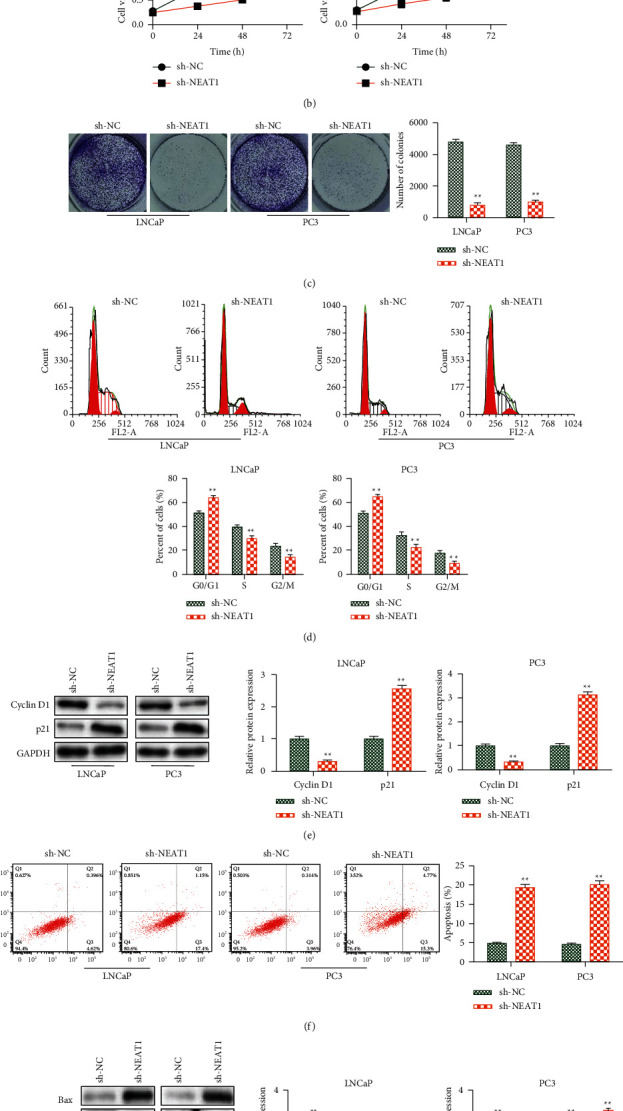
Knockdown of NEAT1 inhibited cell progression in prostate cancer. (a) The level of NEAT1 measured by qRT-PCR. (b) The cell viability detected by the CCK-8 assay. (c) Cell proliferation was tested by colony formation assay. (d) Cell cycle measured by the flow cytometry assay. (e) The cell cycle-related protein detected by western blot analysis. (f) Cell apoptosis assessed by the flow cytometry assay. (g) Cell apoptosis-related protein tested by western blot analysis. ^*∗∗*^*P* < 0.01, sh-NEAT1 vs. sh-NC.

Next, sh-NEAT1 and its control sh-NC were used to treat LNCaP and PC3 cells to explore the functions of NEAT1 in PCa. qRT-PCR analysis showed that sh-NEAT1 transfection markedly inhibited the expression of NEAT1 in LNCaP and PC3 cells (Figure 2(a)). The CCK-8 assay revealed that the cell viability was significantly inhibited by NEAT1 knockdown in LNCaP and PC3 cells (Figure 2(b)). The colony formation assay showed that silencing of NEAT1 decreased the colony number of LNCaP and PC3 cells (Figure 2(c)). Furthermore, flow cytometry analysis detected whether NEAT1 was involved in cell cycle progression. The results indicated that NEAT1 silencing significantly increased the percentage of G0/G1 phase cells and decreased S and G2/M phase cells (Figure 2(d)). Meanwhile, we also found that NEAT1 knockdown decreased cyclin D1 expression and increased p21 expression in LNCaP and PC3 cells (Figure 2(e)). Moreover, flow cytometry analysis and western blot analysis illustrated that NEAT1 knockdown positively affected apoptosis, including increasing apoptosis rate, upregulating Bax, cleaved-caspase-9, and cleaved-caspase-3 expression and decreasing the expression of Bcl-2 (Figures 2(f) and 2(g)).

### 3.3. Downregulation of NEAT1 Repressed Cell Migration and Invasion in PCa

**Figure 3:**
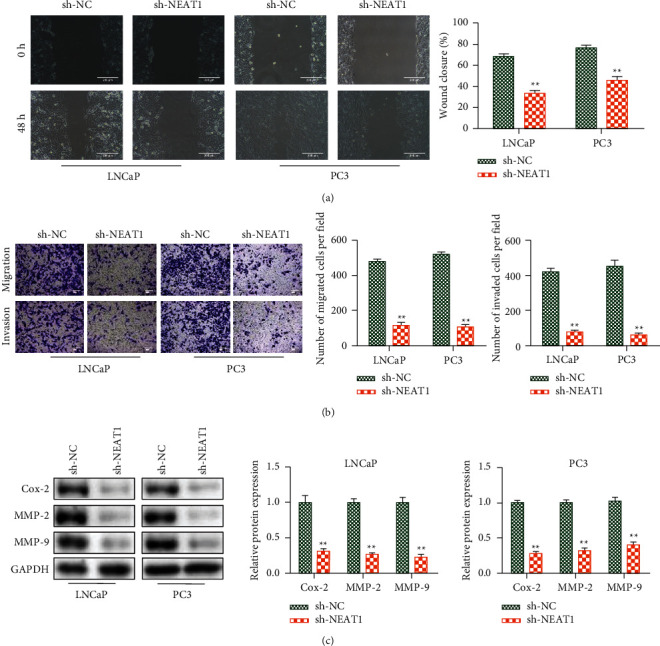
Knockdown of NEAT1 repressed cell migration and invasion in prostate cancer. (a) Cell migration ability was detected by the scratch test in LNCap and PC3 cells (100×). (b) Cell migration and invasion abilities were measured by the transwell assay (100×). (c) Migration- and invasion-related proteins were assessed via western blot analysis. ^*∗∗*^*P* < 0.01, sh-NEAT1 vs. sh-NC.

On the other hand, the scratch test and transwell analysis were used to examine the functions of NEAT1 on cell migration and invasion. The results illustrated that NEAT1 silencing dramatically reduced the migration and invasion in LNCaP and PC3 cells (Figures 3(a) and 3(b)). Consistently, the expressions of migration- and invasion-associated proteins (Cox-2, MMP-2, and MMP-9) were lower in the sh-NEAT1 group compared with the sh-NC group (Figure 3(c)). All these data suggested the inhibition of NEAT1 repressed PCa progression.

### 3.4. NEAT1 Exerts Its Function by Sponging miRNA-766-5p

**Figure 4:**
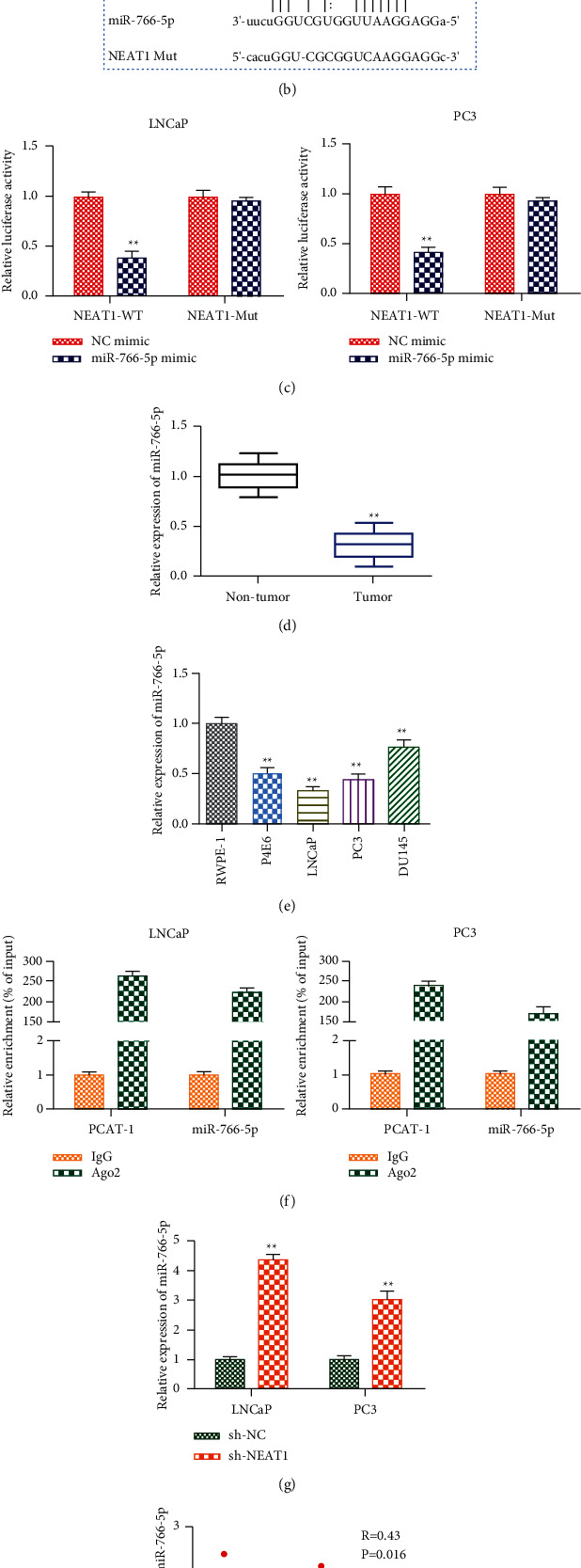
miRNA-766-5p targeted 3′-UTR of NEAT1. (A) The location of NEAT1. ^*∗∗*^*P* < 0.01, nuclear vs. cytoplasmic. (b) The binding sites between NEAT1 and miRNA-766-5p were predicted by StarBase. (c) The relationship between NEAT1 and miRNA-766-5p was testified by the luciferase reporter assay. ^*∗∗*^*P* < 0.01, miR-766-5p mimic vs. NC mimic. (d) The level of miRNA-766-5p in PCa tissues. ^*∗∗*^*P* < 0.01, tumor vs. nontumor. (e) The level of miRNA-766-5p in PCa cell lines. ^*∗∗*^*P* < 0.01, P4E6, LNCaP, PC3, and DU145 vs. RWPE-1. (f) The interaction between NEAT1 and miRNA-766-5p was testified by the RIP assay. ^*∗∗*^*P* < 0.01, Ago2 vs. IgG. (g) The level of miRNA-766-5p in sh-NEAT1-treated cells. ^*∗∗*^*P* < 0.01, sh-NEAT1 vs. sh-NC. (h) Pearson's correlation analysis demonstrated the correlation of NEAT1 expression with miRNA-766-5p.

We detected the cellular position of NEAT1 to investigate the specific mechanism that NEAT1 modulated in PCa. In LNCaP and PC3, NEAT1 was mainly located in the cytoplasm (Figure 4(a)). Since lncRNAs exerted their function by sponging miRNAs, we used the StarBase database to predict the target of NEAT1 and identify miRNA-766-5p and found that there were promising binding sites between NEAT1 and miR‐766‐5p (Figure 4(b)). The luciferase reporter vectors NEAT1-WT or NEAT1-Mut were constructed to transfect into LNCaP and PC3 cells to verify their connection. From the results, miR-766‐5p mimic greatly reduced the luciferase activities of NEAT1-WT but had no effect on NEAT1-Mut (Figure 4(c)). Moreover, miRNA-766-5p was low expressed in PCa tissues and cell lines, especially in LNCaP and PC3 (Figures 4(d) and 4(e)). Additionally, RIP experiments demonstrated that NEAT1 and miRNA-766-5p were largely abundant in Ago2-precipitated RNA-induced silence complexes (Figure 4(f)). Then, we found that sh-NEAT1 highly expressed miRNA-766-5p in LNCaP and PC3, and there was a negative relationship between NEAT1 and miRNA-766-5p in clinical samples (Figures 4(g) and 4(h)). These results suggested that NEAT1 played its roles via sponging miRNA-766-5p.

### 3.5. Upregulation of miRNA-766-5p Inhibited PCa Proliferation, Migration, and Invasion but Promoted Apoptosis

**Figure 5:**
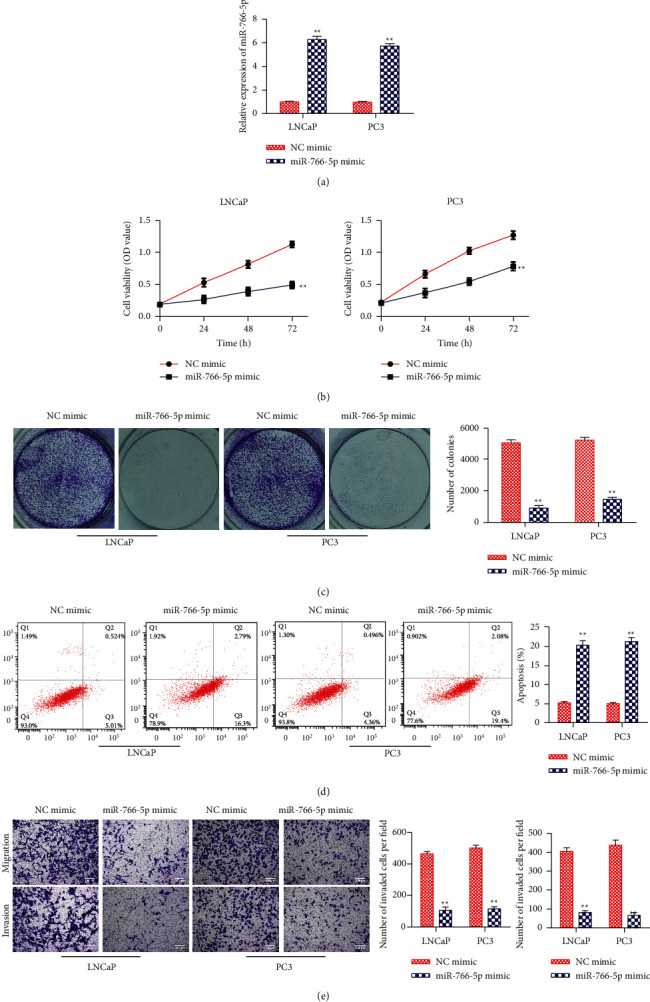
miRNA-766-5p overexpression inhibited PCa proliferation, migration, and invasion. LNCaP and PC3 cells were treated by miRNA-766-5p mimic and NC mimic. (a) The level of miRNA-766-5p. (b) The cell viability was detected via the CCK-8 assay. (c) Cell proliferation was tested via the colony formation assay. (d) Cell apoptosis was measured via the flow cytometry assay. (e) Cell migration and invasion abilities were measured by the transwell assay (100×). ^*∗∗*^*P* < 0.01, miR-766-5p mimic vs. NC mimic.

We next studied the functions of miRNA-766-5p on PCa migration, proliferation, and invasion. Firstly, we tested the overexpression efficiency of miRNA-766-5p, finding that miRNA-766-5p mimic observably promoted miRNA-766-5p expression in LNCaP and PC3 (Figure 5(a)). Then, overexpression of miRNA-766-5p inhibited the proliferation of LNCaP and PC3 evaluated by the CCK-8 assay and colony formation test (Figures 5(b) and 5(c)). Meanwhile, flow cytometry analysis also showed the negative effect of miRNA-766-5p overexpression on cell apoptosis (Figure 5(d)). Furthermore, transwell analysis demonstrated that upregulation of miRNA-766-5p observably inhibited migration and invasion of LNCaP and PC3 (Figure 5(e)).

### 3.6. E2F3 Was a Target Gene of miRNA-766-5p

**Figure 6:**
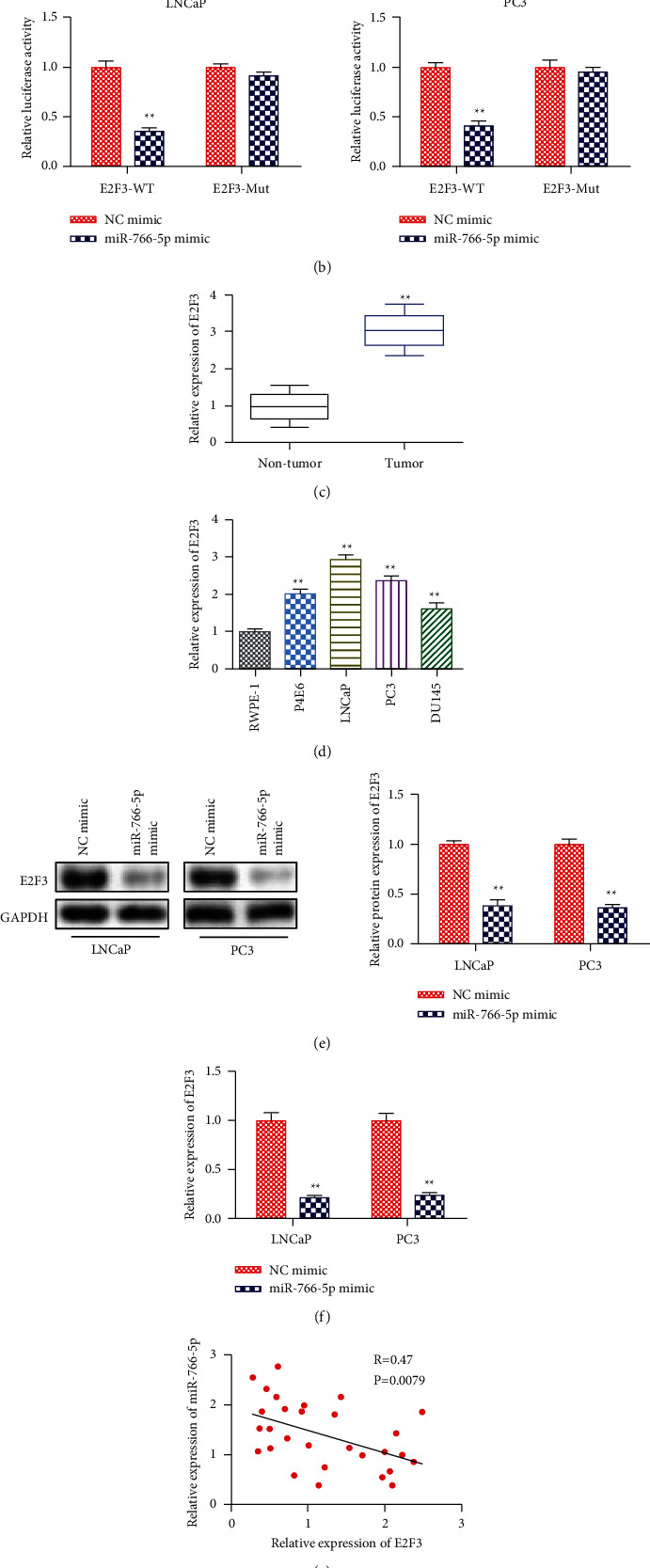
E2F3 was a target gene of miRNA-766-5p. (a) The binding sites between miRNA-766-5p and E2F3 predicted by StarBase. (b) The interaction between miRNA-766-5p and E2F3 testified by the luciferase reporter assay. ^*∗∗*^*P* < 0.01, tumor vs. nontumor. (c) The level of E2F3 in PCa tissues. ^*∗∗*^*P* < 0.01, miR-766-5p mimic vs. NC mimic. (d) The level of E2F3 in PCa cells. ^*∗∗*^*P* < 0.01, P4E6, LNCaP, PC3, and DU145 vs. RWPE-1. (e) The mRNA level of E2F3 in miRNA-766-5p mimic treated cells. ^*∗∗*^*P* < 0.01, miR-766-5p mimic vs. NC mimic. (f) The protein level of E2F3 in miRNA-766-5p mimic treated cells. ^*∗∗*^*P* < 0.01, miR-766-5p mimic vs. NC mimic. (g) Pearson's correlation analysis demonstrated the correlation of miRNA-766-5p expression with E2F3.

To study the downstream functional genes of miRNA-766-5p, the StarBase software was used to predict that E2F3 might be a target gene of miRNA-766-5p (Figure 6(a)) since it has been reported to exert major roles in multiple cancers. A luciferase reporter assay was performed to verify this prediction. We found that in the E2F3-WT group, miRNA-766-5p mimic obviously reduced the luciferase activity in LNCaP and PC3 cells, whereas it did not affect the activity in the E2F3-Mut group (Figure 6(b)). Moreover, E2F3 was highly expressed in PCa tissues and cell lines, especially in LNCaP and PC3 (Figures 6(c) and 6(d)). To further evaluate the relationship between miRNA-766-5p and E2F3, we used miRNA-766-5p mimic to transfect LNCaP and PC3 cells. As shown in Figures 6(e) and 6(f), overexpression of miRNA-766-5p inhibited the protein and mRNA expression of E2F3. Moreover, we found that E2F3 expression was negatively correlated with miRNA-766-5p expression (Figure 6(g)).

### 3.7. NEAT1 Silencing Hindered PCa Progression by miRNA-766-5p/E2F3 Axis

**Figure 7:**
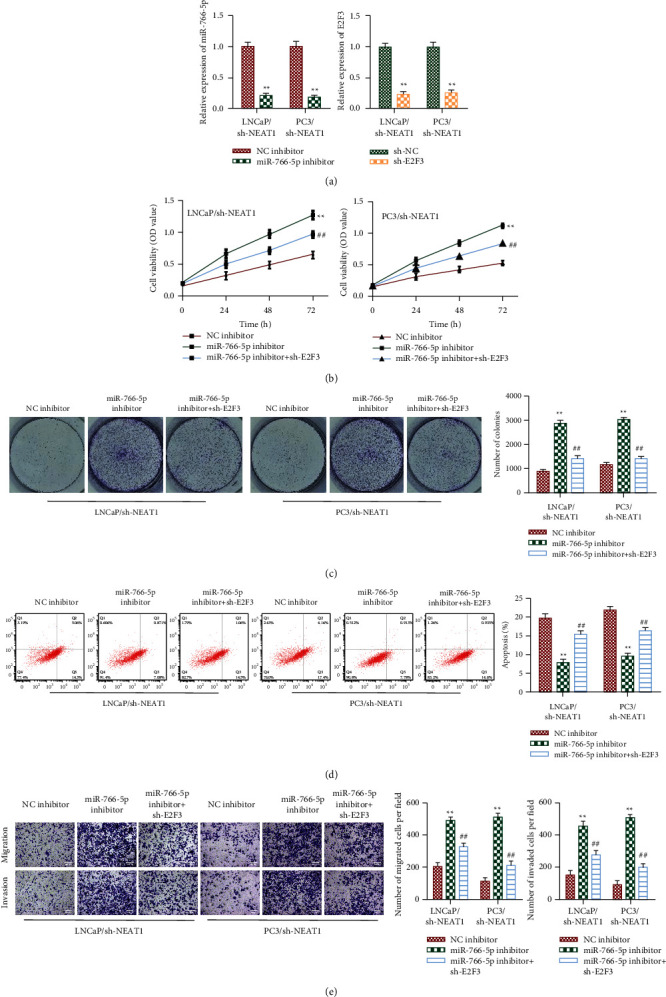
NEAT1 regulated PCa progression by the miRNA-766-5p/E2F3 axis. We constructed stable cell lines via transfecting with sh-NEAT1, followed stimulated with miRNA-766-5p inhibitor and sh-E2F3. (a) The levels of miR-755-5p and E2F3 were detected by qRT-PCR. ^*∗∗*^*P* < 0.01, miR-766-5p inhibitor vs. NC inhibitor, sh-E2F3 vs. sh-NC. (b) Cell viability was determined by CCK-8 analysis. ^*∗∗*^*P* < 0.01, miR-766-5p inhibitor vs. NC inhibitor; ^##^*P* < 0.01, miR-766-5p inhibitor + sh-E2F3 vs. miR-766-5p inhibitor. (c) Cell proliferation was measured by colony formation analysis. ^*∗∗*^*P* < 0.01, miR-766-5p inhibitor vs. NC inhibitor; ^##^*P* < 0.01, miR-766-5p inhibitor + sh-E2F3 vs. miR-766-5p inhibitor. (d) Cell apoptosis was evaluated by flow cytometry analysis. ^*∗∗*^*P* < 0.01, miR-766-5p inhibitor vs. NC inhibitor; ^##^*P* < 0.01, miR-766-5p inhibitor + sh-E2F3 vs. miR-766-5p inhibitor. (e) Cell migration and invasion abilities were measured by the transwell assay (100×). ^*∗∗*^*P* < 0.01, miR-766-5p inhibitor vs. NC inhibitor; ^##^*P* < 0.01, miR-766-5p inhibitor + sh-E2F3 vs. miR-766-5p inhibitor.

At last, we performed rescue assays in sh-NEAT1-transfected LNCaP and PC3. Firstly, qRT-PCR results found that miRNA-766-5p inhibitor downregulated miR-766-5p expression and E2F3 knockdown downregulated the expression of E2F3 (Figure 7(a)). In sh-NEAT1-induced LNCaP and PC3, knockdown of E2F3 dramatically reversed the promotive functions of miRNA-766-5p inhibitor on cell proliferation by CCK-8 and colony formation analysis (Figures 7(b) and 7(c)). In addition, silencing of miRNA-766-5p significantly facilitated apoptosis, inhibited migration and invasion of sh-NEAT1-transfected LNCaP and PC3, but these effects were abrogated by E2F3 silencing (Figures 7(d) and 7(e)). In conclusion, these results indicated that NEAT1 exerted its tumor-promotive functions on PCa by the miRNA-766-5p/E2F3 axis.

## 4. Discussion

Growing evidence has indicated that lncRNAs are involved in various pathologic processes of cancer by regulating gene expressions at epigenetic, transcriptional, and post-transcriptional levels [15]. For PCa oncogenesis, abundant lncRNAs with carcinogenic or anticancer roles have been proved to be involved in this process [16, 17]. In our study, we conducted a series of validation and functional experiments to assess the effect of NEAT1 in the development and occurrence of PCa, finding that knockdown of NEAT1 inhibited PCa tumorigenesis. Simply put, NEAT1 was an oncogenic gene in PCa.

lncRNA NEAT1 (nuclear‐enriched abundant transcript 1) was firstly reported in 2007. It is enriched in the nucleus and is an important component of near-nuclear paraspeckles, regulating gene expression mainly through stabilizing mRNA in the nucleus. NEAT1 is located on chromosome 11q13, with two variants: NEAT1_1 (3.7 kb) and NEAT1_2 (23 kb) [18]. Recently, accumulating evidence suggests that NEAT1 acts as tumor-promoting in the occurrence and development of most tumors, such as breast cancer [19], multiple myeloma [20], and pancreatic cancer [21]. Li et al. found that in hepatocellular carcinoma, NEAT 1, as a sponge for miR-204, suppresses its expression to increase ATG3 expression to promote autophagy and enhances the resistance of the cells to sorafenib [22]. Qi et al. revealed that NEAT1 competes against let-7a to promote cell proliferation and metastasis in non-small cell lung cancer (NSCLC) [23]. Additionally, NEAT1 expression is positively correlated with poor prognosis. Chen et al. reported that NEAT1 expression in esophageal squamous cell carcinoma (ESCC) is significantly upregulated and is closely related to tumor size and clinical staging, suggesting that the high expression of NEAT1 is one of the independent indicators of poor prognosis in patients with ESCC [24].

Although NEAT1 is reported to be the transcriptional target of p53, it plays an important role in the tumor suppressive function of p53 in osteosarcoma, breast cancer, lung cancer, etc. [25, 26]. However, it plays the opposite role in prostate cancer. Wen et al. found that M6A hypermethylation of NEAT1_1 promotes bone metastasis of prostate cancer [27]. NEAT1 drives PCa growth by changing the epigenetic landscape of the target gene promoter to facilitate transcription [28]. Besides, Xiong et al. found that NEAT1 promotes PCa cells growth via modulating the SRC3/IGF1R/AKT axis [29].

In our study, NEAT1 was overexpressed in PCa tissues and cells, which is consistent with the previous result [30]. NEAT1 silencing suppressed the migration, proliferation, and invasion of PCa. Hence, NEAT1 was a cancer-promoting gene in PCa.

Increasing reports have exhibited that miRNAs participate in numerous biological behaviors of tumor progression, including invasion and apoptosis [31]. Lots of research showed that lncRNA took effects on tumorigenesis via regulation of specific miRNA. In our research, we first discovered that NEAT1 was mainly distributed in the cytoplasm of PCa cells through subcellular separation and qRT-PCR analysis, revealing that NEAT1 might regulate the expression of its downstream genes after transcription. Subsequently, bioinformatics prediction and luciferase reporter gene analysis determined that NEAT1 served as a ceRNA for miRNA-766-5p and negatively regulated miRNA-766-5p expression. miRNA-766-5p has been reported to exert major roles in multiple cancers, such as lung adenocarcinoma [32], colorectal cancer [33], and glioma [34]. The present study further verified that miRNA-766-5p expression was greatly downregulated in PCa tissues and cells, and miRNA-766-5p overexpression restrained the migration, proliferation, and invasion.

Next, we further explored the molecular mechanisms of miRNA-766-5p in PCa. Using StarBase websites, we predicted the target gene of miRNA-766-5p and identified E2F3. E2F3, located on chromosome 6p22, has been shown to be significantly upregulated in cancers and is associated with malignancy progression, including proliferation and cell cycle process [35, 36]. In this study, E2F3 was positively correlated with NEAT1 whereas was negatively correlated with miRNA-766-5p. Also, rescue experiments demonstrated that E2F3 overexpression reversed that miRNA-766-5p inhibitor-induced positive function on the proliferation, migration, and invasion in the sh-NEAT1 stable cell line. However, the present study also has some limitations. The main limitation is the lack of *in vivo* experiments. Therefore, a complete *in vivo* experiment should be employed to verify our results.

In conclusion, this study demonstrated the main roles of the NEAT1/miRNA-766-5p/E2F3 axis in the proliferation, migration, and invasion of PCa cells. NEAT1 promoted PCa cell proliferation, migration, and invasion via sponging miR-776-5p and consequently increased E2F3 expression, suggesting that NEAT1 might be a potential therapeutic target for PCa.

## Figures and Tables

**Table 1:** Primer sequences used for qRT-PCR.

Genes		Primer sequences (5′–3′)
NEAT1	Forward	AGGCAGGGAGAGGTAGAAGG
	Reverse	TGGCATGGACAAGTTGAAGA
GAPDH	Forward	GCTTCGGCAGCACATATACTAAAAT
	Reverse	CGCTTCACGAATTTGCGTGTCAT
E2F3	Forward	CAGGCTGGTTTCGGAAATGC
	Reverse	TGGACTTCGTAGTGCAGCTC
miRNA-766-5p	Forward	TAAAATAGGAGTACTGTCTAA
	Reverse	ATTAGTAAATTGGCTGCTGCAG
U6	Forward	CTCGCTTCGGCAGCACATATACTA
	Reverse	ACGAATTTGCGTGTCATCCTTGCG

## Data Availability

Data archiving will be made available on reasonable request. Runyuan Ji is responsible for the data.

## Supplementary Materials

Supplementary Materials Figure S1: the NEAT1 expression profile analyzed by the GEO database. The original data of GSE29079 were downloaded and analyzed by GEO2R.

## References

[1] Litwin M. S., Tan H.-J. (2017). The diagnosis and treatment of prostate cancer. *JAMA*.

[2] Siegel R. L., Miller K. D., Jemal A. (2019). Cancer statistics, 2019. *CA: A Cancer Journal for Clinicians*.

[3] Liu X., Yu C., Bi Y., Zhang Z. J. (2019). Trends and age-period-cohort effect on incidence and mortality of prostate cancer from 1990 to 2017 in China. *Public Health*.

[4] Poorthuis M. H. F., Vernooij R. W. M., van Moorselaar R. J. A., de Reijke T. M. (2017). Second-line therapy in patients with metastatic castration-resistant prostate cancer with progression after or under docetaxel: a systematic review of nine randomized controlled trials. *Seminars in Oncology*.

[5] Chen R., Sheng L., Zhang H.-J., Ji M., Qian W.-Q. (2018). miR-15b-5p facilitates the tumorigenicity by targeting RECK and predicts tumour recurrence in prostate cancer. *Journal of Cellular and Molecular Medicine*.

[6] Cao H., Wahlestedt C., Kapranov P. (2018). Strategies to annotate and characterize long noncoding RNAs: advantages and pitfalls. *Trends in Genetics*.

[7] Peng W.-X., Koirala P., Mo Y.-Y. (2017). LncRNA-mediated regulation of cell signaling in cancer. *Oncogene*.

[8] Huarte M. (2015). The emerging role of lncRNAs in cancer. *Nature Medicine*.

[9] Su Y., Wu H., Pavlosky A. (2016). Regulatory non-coding RNA: new instruments in the orchestration of cell death. *Cell Death & Disease*.

[10] Fatica A., Bozzoni I. (2014). Long non-coding RNAs: new players in cell differentiation and development. *Nature Reviews Genetics*.

[11] Olgun G., Sahin O., Tastan O. (2018). Discovering lncRNA mediated sponge interactions in breast cancer molecular subtypes. *BMC Genomics*.

[12] Luo Z. F., Peng Y., Liu F. H. (2020). Long noncoding RNA SNHG14 promotes malignancy of prostate cancer by regulating with miR-5590-3p/YY1 axis. *European Review for Medical and Pharmacological Sciences*.

[13] Long B., Li N., Xu X.-X. (2018). Long noncoding RNA LOXL1-AS1 regulates prostate cancer cell proliferation and cell cycle progression through miR-541-3p and CCND1. *Biochemical and Biophysical Research Communications*.

[14] Xu T., Liu C. L., Li T., Zhang Y. H., Zhao Y. H. (2019). LncRNA TUG1 aggravates the progression of prostate cancer and predicts the poor prognosis. *European Review for Medical and Pharmacological Sciences*.

[15] Xiao M., Feng Y., Liu C., Zhang Z. (2018). Prognostic values of long noncoding RNA PVT1 in various carcinomas: an updated systematic review and meta-analysis. *Cell Proliferation*.

[16] Hua J. T., Guo H., Zhang Y. (2018). Risk SNP-mediated promoter-enhancer switching drives prostate cancer through lncRNA PCAT19. *Cell*.

[17] Ren S., Peng Z., Mao J.-H. (2012). RNA-seq analysis of prostate cancer in the Chinese population identifies recurrent gene fusions, cancer-associated long noncoding RNAs and aberrant alternative splicings. *Cell Research*.

[18] Clemson C. M., Hutchinson J. N., Sara S. A. (2009). An architectural role for a nuclear noncoding RNA: NEAT1 RNA is essential for the structure of paraspeckles. *Molecular Cell*.

[19] Li X., Wang S., Li Z. (2017). The lncRNA NEAT1 facilitates cell growth and invasion via the miR-211/HMGA2 axis in breast cancer. *International Journal of Biological Macromolecules*.

[20] Taiana E., Favasuli V., Ronchetti D. (2020). Long non-coding RNA NEAT1 targeting impairs the DNA repair machinery and triggers anti-tumor activity in multiple myeloma. *Leukemia*.

[21] Feng Y., Gao L., Cui G., Cao Y. (2020). LncRNA NEAT1 facilitates pancreatic cancer growth and metastasis through stabilizing ELF3 mRNA. *American journal of cancer research*.

[22] Li X., Zhou Y., Yang L. (2020). LncRNA NEAT1 promotes autophagy via regulating miR‐204/ATG3 and enhanced cell resistance to sorafenib in hepatocellular carcinoma. *Journal of Cellular Physiology*.

[23] Qi L., Liu F., Zhang F. (2018). lncRNA NEAT1 competes against let-7a to contribute to non-small cell lung cancer proliferation and metastasis. *Biomedicine & Pharmacotherapy*.

[24] Chen X., Kong J., Ma Z., Gao S., Feng X. (2015). Up regulation of the long non-coding RNA NEAT1 promotes esophageal squamous cell carcinoma cell progression and correlates with poor prognosis. *American journal of cancer research*.

[25] Idogawa M., Ohashi T., Sasaki Y., Nakase H., Tokino T. (2017). Long non‐coding RNA NEAT1 is a transcriptional target of p53 and modulates p53‐induced transactivation and tumor‐suppressor function. *International Journal of Cancer*.

[26] Adriaens C., Standaert L., Barra J. (2016). p53 induces formation of NEAT1 lncRNA-containing paraspeckles that modulate replication stress response and chemosensitivity. *Nature Medicine*.

[27] Wen S., Wei Y., Zen C., Xiong W., Niu Y., Zhao Y. (2020). Long non-coding RNA NEAT1 promotes bone metastasis of prostate cancer through N6-methyladenosine. *Molecular Cancer*.

[28] Chakravarty D., Sboner A., Nair S. S. (2014). The oestrogen receptor alpha-regulated lncRNA NEAT1 is a critical modulator of prostate cancer. *Nature Communications*.

[29] Xiong W., Huang C., Deng H. (2018). Oncogenic non-coding RNA NEAT1 promotes the prostate cancer cell growth through the SRC3/IGF1R/AKT pathway. *The International Journal of Biochemistry & Cell Biology*.

[30] Idogawa M., Nakase H., Sasaki Y., Tokino T. (2019). Prognostic effect of long noncoding RNA NEAT1 expression depends on p53 mutation status in cancer. *Journal of oncology*.

[31] Acunzo M., Romano G., Wernicke D., Croce C. M. (2015). MicroRNA and cancer - a brief overview. *Advances in Biological Regulation*.

[32] Wang M., Liao Q., Zou P. (2020). PRKCZ-AS1 promotes the tumorigenesis of lung adenocarcinoma via sponging miR-766-5p to modulate MAPK1. *Cancer Biology & Therapy*.

[33] Jia B., Xia L., Cao F. (2018). The role of miR-766-5p in cell migration and invasion in colorectal cancer. *Experimental and Therapeutic Medicine*.

[34] Mao Y., Shen G., Su Z., Du J., Xu F., Yu Y. (2020). RAD21 inhibited transcription of tumor suppressor MIR4697HG and led to glioma tumorigenesis. *Biomedicine & Pharmacotherapy*.

[35] van den Heuvel S., Dyson N. J. (2008). Conserved functions of the pRB and E2F families. *Nature Reviews Molecular Cell Biology*.

[36] Zang H., Li Y., Zhang X., Huang G. (2020). Circ‐RNF111 contributes to paclitaxel resistance in breast cancer by elevating E2F3 expression via miR ‐140‐5p. *Thoracic Cancer*.

