# Supplementary material for: The lncRNA NEAT1/miRNA-766-5p/E2F3 Regulatory Axis Promotes Prostate Cancer Progression
Source: J Oncol. 2022 Feb 21;2022:1866972. doi: 10.1155/2022/1866972 (PMC8885187; doi:10.1155/2022/1866972)
Supplement: Supplementary Materials — Figure S1: the NEAT1 expression profile analyzed by the GEO database. The original data of GSE29079 were downloaded and analyzed by GEO2R. [file 1866972.f1.docx]

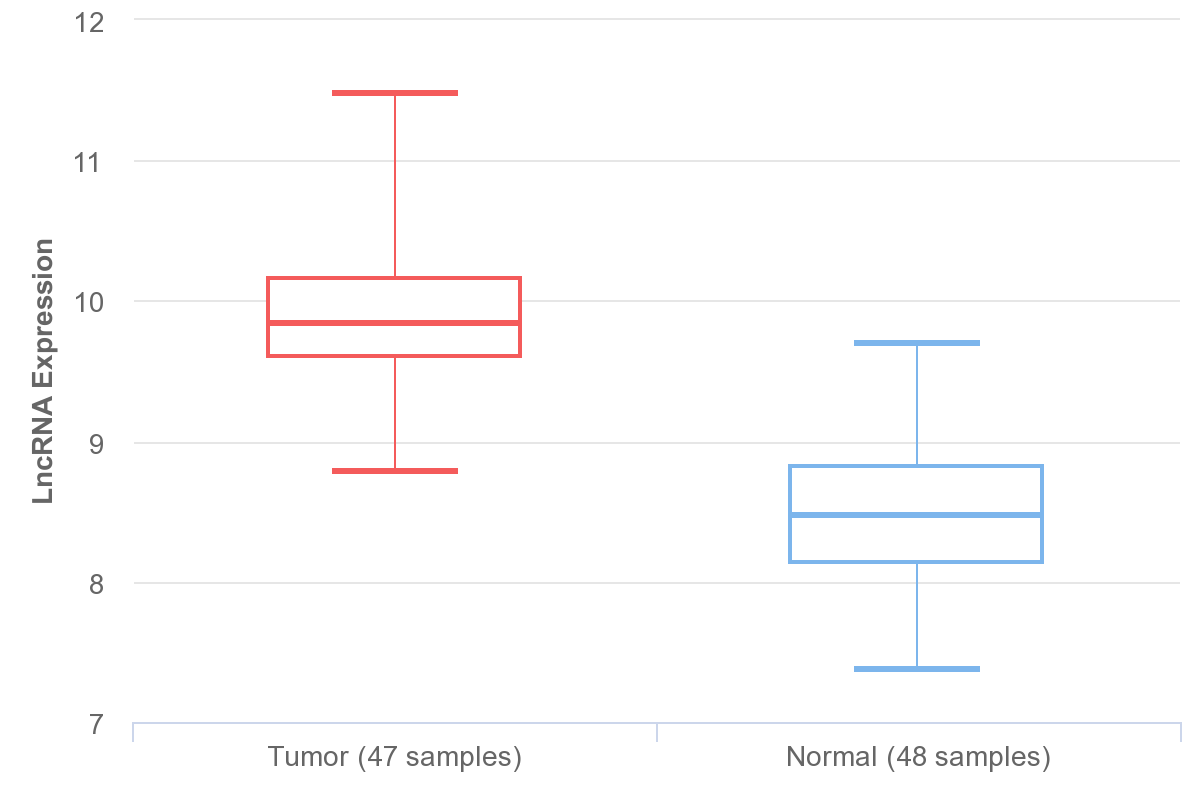


**Figure S1. The NEAT1 expression profile analyzed by GEO database.** The original data of GSE29079 were downloaded and analyzed by GEO 2R.
